# Antioxidant, Antiapoptotic, and Anti-Inflammatory Effects of Hesperetin in a Mouse Model of Lipopolysaccharide-Induced Acute Kidney Injury

**DOI:** 10.3390/molecules28062759

**Published:** 2023-03-18

**Authors:** Ah Young Yang, Hye Jin Choi, Kiryeong Kim, Jaechan Leem

**Affiliations:** Department of Immunology, School of Medicine, Daegu Catholic University, Daegu 42472, Republic of Korea

**Keywords:** hesperetin, lipopolysaccharide, acute kidney injury

## Abstract

Sepsis is a severe inflammatory condition that can cause organ dysfunction, including acute kidney injury (AKI). Hesperetin is a flavonoid aglycone that has potent antioxidant and anti-inflammatory properties. However, the effect of hesperetin on septic AKI has not yet been fully investigated. This study examined whether hesperetin has a renoprotective effect on lipopolysaccharide (LPS)-induced septic AKI. Hesperetin treatment ameliorated histological abnormalities and renal dysfunction in LPS-injected mice. Mechanistically, hesperetin attenuated LPS-induced oxidative stress, as evidenced by the suppression of lipid and DNA oxidation. This beneficial effect of hesperetin was accompanied by downregulation of the pro-oxidant NADPH oxidase 4, restoration of glutathione levels, and activation of antioxidant enzymes. This flavonoid compound also inhibited apoptotic cell death via suppression of p53-dependent caspase-3 pathway. Furthermore, hesperetin alleviated Toll-like receptor 4-mediated cytokine production and macrophage infiltration. Our findings suggest that hesperetin ameliorates LPS-induced renal structural and functional injury through suppressing oxidative stress, apoptosis, and inflammation.

## 1. Introduction

Sepsis is a severe inflammatory condition caused by microbial pathogens, including bacteria, viruses, and fungi [1]. The incidence of sepsis is steadily increasing, adding to social and economic burdens [2]. Sepsis can cause organ dysfunction and is a principal cause of death in critically ill patients. Acute kidney injury (AKI) is a common complication in septic patients, and about 60% of septic patients develop AKI [3]. Indeed, sepsis is a leading cause of AKI in critically ill patients and accounts for 45–70% of all AKI cases [3]. It has also well known that AKI is associated with an increased risk of developing chronic kidney disease [4]. Current therapies for patients with septic AKI include antibiotics, fluid therapy, and vasopressors, but there is no specific treatment for the disease [3]. Therefore, the development of effective and specific therapeutic strategies for septic AKI is urgently needed.

Among the microbial pathogens, Gram-negative bacteria (GNB) are known to be frequently involved in septic AKI [5]. Lipopolysaccharide (LPS) is the major structural component of the membrane of GNB [6]. LPS is recognized by Toll-like receptor 4 (TLR4), which is expressed on renal cells, such as tubular epithelial cells, as well as immune cells, such as macrophages [7]. Upon activation, TLR4 mediates its function in the inflammatory response by recruiting the cytoplasmic protein, myeloid differentiation primary response 88 (MyD88) [7]. During Gram-negative bacteremia, the stimulation of TLR4 by LPS in immune cells leads to the overproduction of proinflammatory cytokines and reactive oxygen species (ROS) as well as the infiltration of immune cells into the damaged organ [8,9]. Exposure of LPS to tubular epithelial cells also induces ROS production and apoptotic cell death [10,11]. Eventually, these changes result in structural and functional renal injury. As such, because of the essential role of LPS in the pathophysiology of septic AKI, rodent models of LPS injection are widely used to study mechanisms of sepsis-induced organ dysfunction and discover new therapies [12].

Hesperetin is a natural flavonoid that has antioxidant and anti-inflammatory properties [13,14]. Administration of hesperetin can ameliorate myocardial ischemia [15], calcific aortic valve disease [16], fatty liver disease [17], osteoarthritis [18], colitis [19], acute lung injury [20], and Alzheimer’s disease [21] in rodents. In addition, hesperetin can alleviate unilateral ureteral obstruction-induced renal injury and fibrosis through inhibiting epithelial–mesenchymal transition in mice [22]. The renoprotective effect of hesperetin was also observed in an animal model of diabetic nephropathy [23]. Furthermore, hesperetin ameliorates cisplatin-induced AKI via inhibition of oxidative stress, inflammation, and apoptosis in rodents [24,25]. However, whether hesperetin has a protective effect against septic AKI remains undetermined. Therefore, the present study investigated the effect and mechanism of hesperetin on LPS-induced AKI.

## 2. Results

### 2.1. Hesperetin Ameliorated LPS-Induced Structural Injury

**Figure 1:**
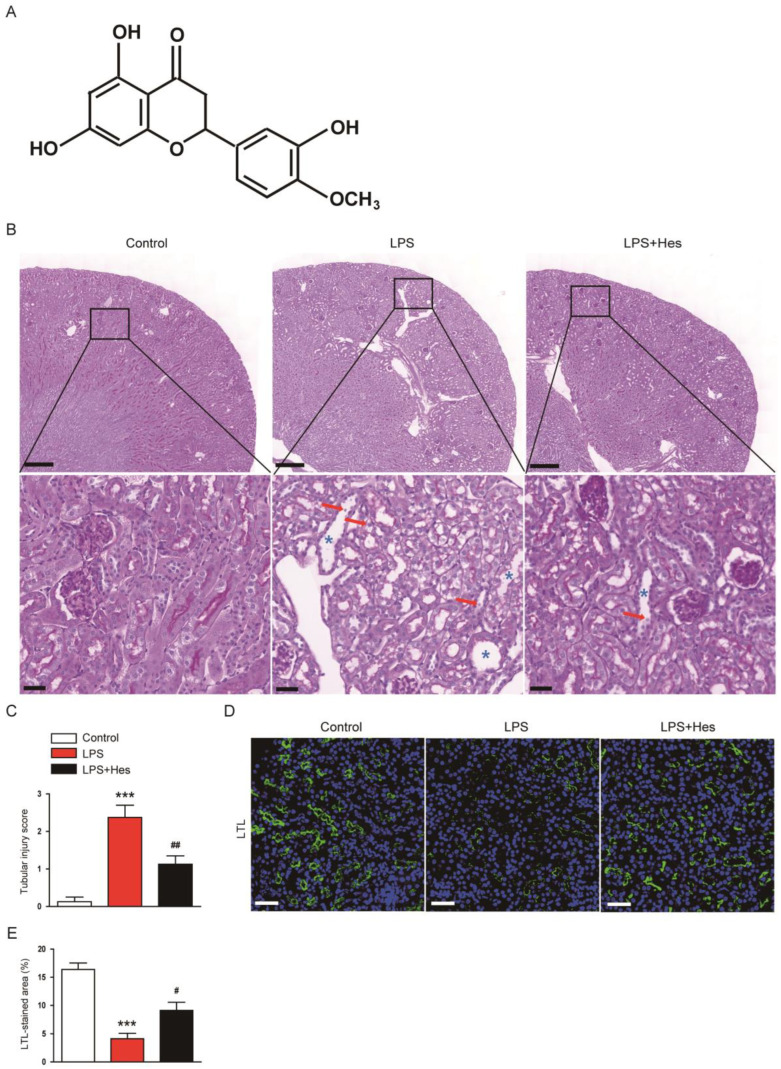
Hesperetin ameliorated histological abnormalities in lipopolysaccharide (LPS)-injected mice. (**A**) The chemical structure of hesperetin. (**B**) Periodic acid-Schiff (PAS) staining. Red arrows indicate detached tubular cells. Blue asterisks indicate dilated tubules. Scale bars: 300 μm (upper panel) and 30 μm (lower panel). (**C**) Tubular injury score. (**D**) Lotus tetragonolobus lectin (LTL) staining (green). Nuclei were stained with 4′,6-diamidino-2-phenylindole (DAPI; blue). Scale bar: 50 μm. (**E**) Percentage of the LTL-stained area per field. *n* = 8 per group. *** *p* < 0.001 vs. Control. ^#^ *p* < 0.05 and ^##^ *p* < 0.01 vs. LPS.

To evaluate the effect of hesperetin (Figure 1A) on LPS-induced histological alterations, periodic acid-Schiff (PAS) staining was performed on sections. LPS-injected mice exhibited marked histological abnormalities including tubular cell detachment and tubular dilatation (Figure 1B,C). However, hesperetin treatment alleviated LPS-induced renal tubular structural damages (tubular injury score: LPS, 2.4 ± 0.3 vs. LPS+Hes, 1.1 ± 0.2, *p* < 0.01; Figure 1B,C). Next, the brush border of proximal tubules was visualized using a fluorescein isothiocyanate (FITC)-conjugated lotus tetragonolobus lectin (LTL) to examine the brush border loss [26,27]. Immunofluorescence (IF) staining revealed that LPS injection reduced the percentage of the LTL-stained area, but this change was mitigated by hesperetin (LPS, 4.1 ± 1.0% vs. LPS+Hes, 9.1 ± 1.4%, *p* < 0.05; Figure 1D,E).

**Figure 2:**
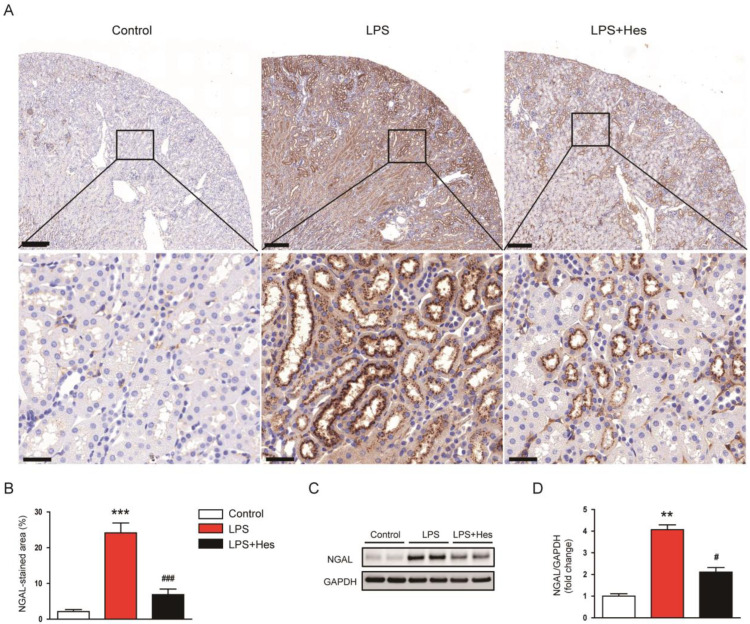
Hesperetin decreased renal neutrophil gelatinase-associated lipocalin (NGAL) expression in LPS-injected mice. (**A**) Immunohistochemistry (IHC) staining for NGAL. Scale bars: 200 μm (upper panel) and 30 μm (lower panel). (**B**) Percentage of the NGAL-stained area per field. (**C**) Western blotting of NGAL. (**D**) Densitometric analysis of bands in (**C**). *n* = 8 per group. ** *p* < 0.01 and *** *p* < 0.001 vs. Control. ^#^ *p* < 0.05 and ^###^ *p* < 0.001 vs. LPS.

To further confirm the protective effect of hesperetin on tubular injury, sections were stained with an antibody against neutrophil gelatinase-associated lipocalin (NGAL), an established tubular injury marker [28]. Immunohistochemistry (IHC) staining showed that LPS increased the percentage of the NGAL-stained area (Figure 2A,B). However, hesperetin treatment significantly decreased NGAL expression (LPS, 24.1 ± 2.8% vs. LPS+Hes, 6.9 ± 1.6%, *p* < 0.001; Figure 2A,B). Western blot analysis confirmed the suppressive action of hesperetin on NGAL expression (LPS, 4.1 ± 0.2 vs. LPS+Hes, 2.1 ± 0.2, *p* < 0.05; Figure 2C,D).

### 2.2. Hesperetin Attenuated LPS-Induced Renal Dysfunction

**Figure 3:**
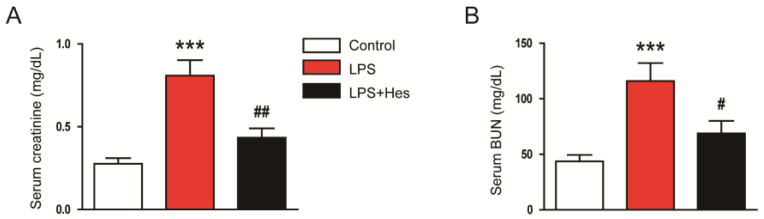
Hesperetin attenuated renal dysfunction in LPS-injected mice. (**A**) Serum creatinine. (**B**) Serum blood urea nitrogen (BUN). *n* = 8 per group. *** *p* < 0.001 vs. Control. ^#^ *p* < 0.05 and ^##^ *p* < 0.01 vs. LPS.

To assess renal function, serum creatinine and blood urea nitrogen (BUN) levels were analyzed [29]. Serum levels of these indicators were elevated after LPS injection but were attenuated by hesperetin (creatinine: LPS, 0.81 ± 0.09 mg/dL vs. LPS+Hes, 0.43 ± 0.06 mg/dL, *p* < 0.01; BUN: LPS, 115.9 ± 16.3 mg/dL vs. LPS+Hes, 68.9 ± 11.2 mg/dL, *p* < 0.05; Figure 3A,B).

### 2.3. Hesperetin Inhibited LPS-Induced Oxidative Stress

**Figure 4:**
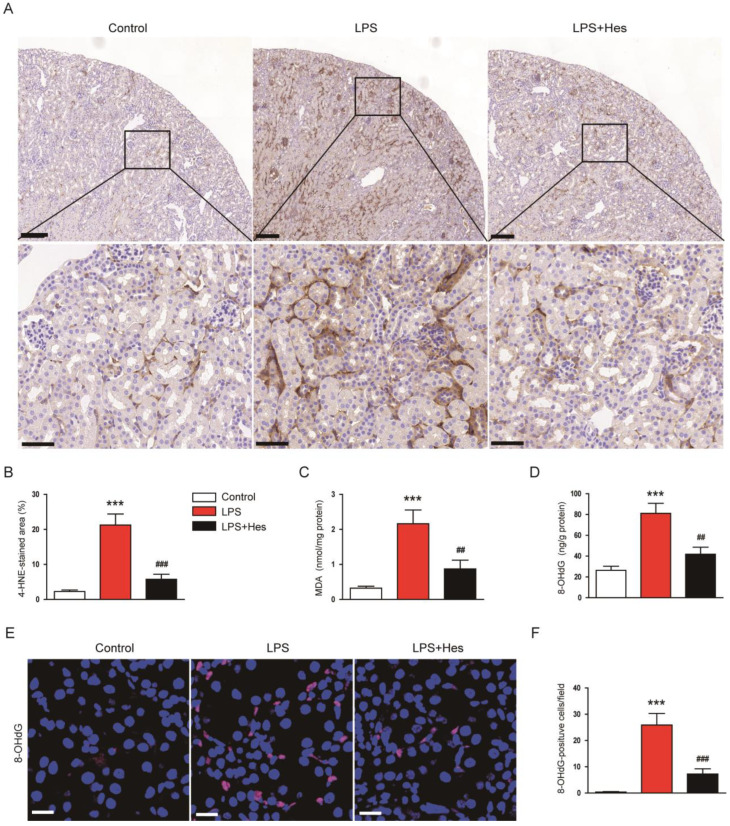
Hesperetin inhibited LPS-induced oxidative stress. (**A**) IHC staining for 4-hydroxynonenal (4-HNE). Scale bars: 150 μm (upper panel) and 50 μm (lower panel). (**B**) Percentage of the 4-HNE-stained area per field. (**C**) Renal malondialdehyde (MDA) levels. (**D**) Renal 8-hydroxy-2′-deoxyguanosine (8-OHdG) levels. (**E**) IF staining for 8-OHdG. Scale bar: 20 μm. (**F**) Number of 8-OHdG-positive cells per field. *n* = 8 per group. *** *p* < 0.001 vs. Control. ^##^ *p* < 0.01 and ^###^ *p* < 0.001 vs. LPS.

Numerous studies suggest that oxidative stress is a key pathological process in septic AKI [8,9]. Therefore, we performed IHC staining for 4-hydroxynonenal (4-HNE), a lipid peroxidation product [30,31], to assess oxidative stress. LPS-injected mice displayed a marked increase in the 4-HNE-stained area (Figure 4A,B). However, hesperetin treatment decreased 4-HNE expression (LPS, 21.3 ± 3.2% vs. LPS+Hes, 5.8 ± 1.5%, *p* < 0.001; Figure 4A,B). Renal levels of malondialdehyde (MDA), another lipid peroxidation product [30,31], were also decreased with hesperetin (LPS, 2.2 ± 0.4 nmol/mg protein vs. LPS+Hes, 0.9 ± 0.2 nmol/mg protein, *p* < 0.01; Figure 4C). Furthermore, we measured renal 8-hydroxy-2′-deoxyguanosine (8-OHdG) levels as a marker for DNA oxidation [32]. Hesperetin significantly reduced renal 8-OHdG levels (LPS, 81.1 ± 9.6 ng/g protein vs. LPS+Hes, 41.9 ± 6.7 ng/g protein, *p* < 0.01; Figure 4D). IF staining also showed that the number of 8-OHdG-positive cells was significantly reduced by hesperetin (LPS, 25.9 ± 4.4 vs. LPS+Hes, 7.3 ± 2.0, *p* < 0.001; Figure 4E,F).

**Figure 5:**
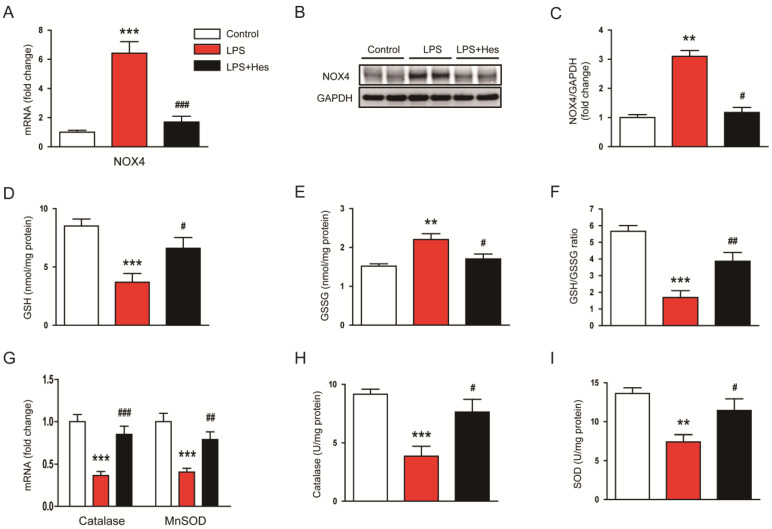
Hesperetin regulated pro-oxidant and antioxidant enzymes. (**A**) Renal mRNA expression of NADPH oxidase 4 (NOX4). (**B**) Western blotting of NOX4. (**C**) Densitometric analysis of bands in (**B**). (**D**) Renal levels of reduced glutathione (GSH). (**E**) Renal levels of oxidized glutathione (GSSG). (**F**) GSH/GSSG ratios. (**G**) Renal mRNA expression of catalase and manganese superoxide dismutase (MnSOD). (**H**) Catalase activity. (**I**) SOD activity. *n* = 8 per group. ** *p* < 0.01 and *** *p* < 0.001 vs. Control. ^#^ *p* < 0.05, ^##^ *p* < 0.01 and ^###^ *p* < 0.001 vs. LPS.

Oxidative stress can occur through an imbalance in pro-oxidant/antioxidant homeostasis [33]. Therefore, to explore the mechanism of hespertin’s inhibitory effect on LPS-induced oxidative stress, we next investigated the expression of pro-oxidant and antioxidant enzymes. LPS-injected mice exhibited increased mRNA expression of the pro-oxidant NADPH oxidase 4 (NOX4) enzyme (Figure 5A). However, hesperetin treatment significantly reduced NOX4 expression (LPS, 6.4 ± 0.8 vs. LPS+Hes, 1.7 ± 0.4, *p* < 0.001; Figure 5A). NOX4 protein levels were also reduced by hesperetin (LPS, 3.1 ± 0.2 vs. LPS+Hes, 1.2 ± 0.2, *p* < 0.05; Figure 5B,C). Moreover, hesperetin increased the amount of the endogenous antioxidant glutathione (GSH) (LPS, 3.7 ± 0.8 nmol/mg protein vs. LPS+Hes, 6.6 ± 0.9 nmol/mg protein, *p* < 0.05; Figure 5D) and the ratio of reduced GSH to oxidized GSH (GSH/GSSG; LPS, 1.7 ± 0.4 vs. LPS+Hes, 3.9 ± 0.5, *p* < 0.01; Figure 5F) in LPS-injected mice, while decreasing the amount of GSSG (LPS, 2.2 ± 0.2 nmol/mg protein vs. LPS+Hes, 1.7 ± 0.1 nmol/mg protein, *p* < 0.05; Figure 5E). LPS injection reduced catalase and manganese superoxide dismutase (MnSOD) mRNA levels, but they were markedly increased by hesperetin (catalase: LPS, 0.37 ± 0.05 vs. LPS+Hes, 0.85 ± 0.10, *p* < 0.001; MnSOD: LPS, 0.41 ± 0.04 vs. LPS+Hes, 0.79 ± 0.09, *p* < 0.01; Figure 5G). Catalase and SOD activities were also increased by hesperetin (catalase: LPS, 3.9 ± 0.8 U/mg protein vs. LPS+Hes, 7.6 ± 1.1 U/mg protein, *p* < 0.05; SOD: LPS, 7.4 ± 0.9 U/mg protein vs. LPS+Hes, 11.5 ± 1.5 U/mg protein, *p* < 0.05; Figure 5H,I).

### 2.4. Hesperetin Alleviated LPS-Induced Apoptosis

**Figure 6:**
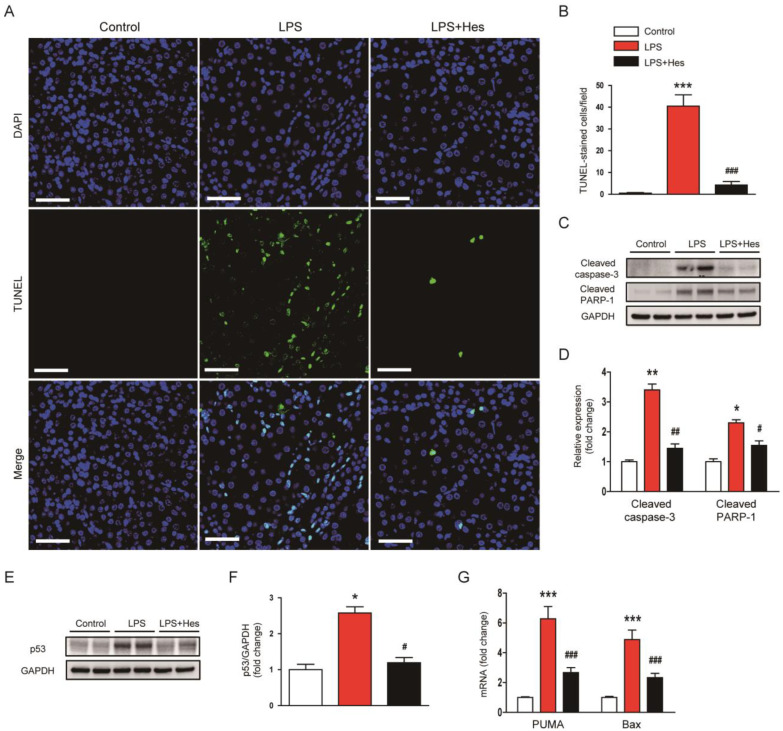
Hesperetin alleviated apoptotic cell death in LPS-injected mice. (**A**) TdT-mediated dUTP nick end labeling (TUNEL) staining (green). Nuclei were stained with DAPI (blue). Scale bar: 50 μm. (**B**) Number of TUNEL-positive cells per field. (**C**) Western blotting of cleaved caspase-3 and cleaved poly(ADP-ribose) polymerase-1 (PARP-1). (**D**) Densitometric analysis of bands in (**C**). (**E**) Western blotting of p53. (**F**) Densitometric analysis of bands in (**E**). (**G**) Renal mRNA expression of p53 upregulated modulator of apoptosis (PUMA) and Bax. *n* = 8 per group. * *p* < 0.05, ** *p* < 0.01 and *** *p* < 0.001 vs. Control. ^#^ *p* < 0.05, ^##^ *p* < 0.01 and ^###^ *p* < 0.001 vs. LPS.

As apoptosis also plays an important role in septic AKI [8,9], we evaluated the effect of hesperetin on apoptosis using TdT-mediated dUTP nick end labeling (TUNEL) assay. LPS-injected mice displayed a higher number of TUNEL-positive cells (Figure 6A,B). However, these changes were mitigated by hesperetin (LPS, 40.5 ± 5.2 vs. LPS+Hes, 4.3 ± 1.6, *p* < 0.001; Figure 6A,B). Further, hesperetin reduced the protein levels of cleaved caspase-3 and cleaved poly(ADP-ribose) polymerase-1 (PARP-1) (cleaved caspase-3: LPS, 3.4 ± 0.2 vs. LPS+Hes, 1.5 ± 0.2, *p* < 0.01; cleaved PARP-1: LPS, 2.3 ± 0.1 vs. LPS+Hes, 1.6 ± 0.2, *p* < 0.05; Figure 6C,D). The expression of p53 transcription factor was also significantly decreased by hesperetin (LPS, 2.6 ± 0.2 vs. LPS+Hes, 1.2 ± 0.1, *p* < 0.05; Figure 6E,F). These changes were accompanied by reduced mRNA levels of its target genes, p53 upregulated modulator of apoptosis (PUMA) and Bax (Figure 6G).

### 2.5. Hesperetin Mitigated LPS-Induced Inflammatory Responses

**Figure 7:**
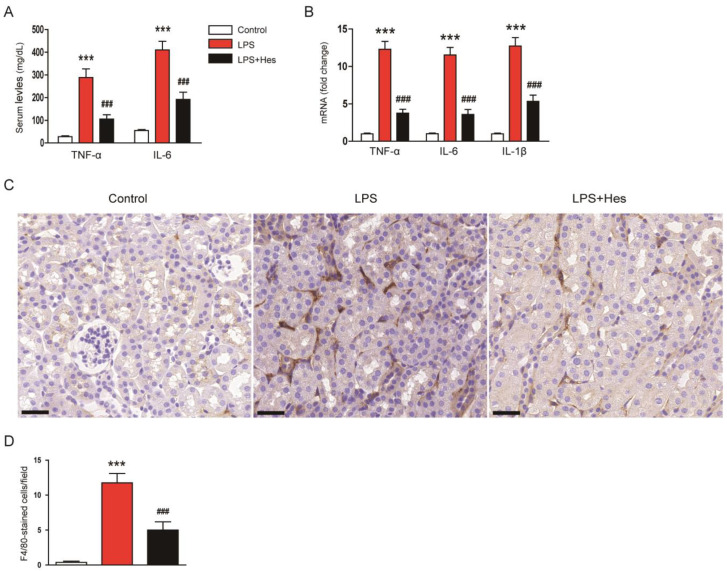
Hesperetin mitigated LPS-induced inflammatory responses. (**A**) Serum tumor necrosis factor-α (TNF-α) and interleukin-6 (IL-6) levels. (**B**) Renal mRNA expression of TNF-α, IL-6, and IL-1β. (**C**) IHC staining for F4/80. Scale bar: 30 μm. (**D**) Number of F4/80-positive cells per field. *n* = 8 per group. *** *p* < 0.001 vs. Control. ^###^ *p* < 0.001 vs. LPS.

LPS induces inflammatory responses that cause organ damage [8,9]. Therefore, the effect of hesperetin on systemic inflammation was investigated by measuring serum levels of tumor necrosis factor-α (TNF-α) and interleukin-6 (IL-6). LPS injection increased serum levels of these cytokines, but they were reduced by hesperetin (TNF-α: LPS, 288.4 ± 38.0 mg/dL vs. LPS+Hes, 105.0 ± 19.2 mg/dL, *p* < 0.001; IL-6: LPS, 410.4 ± 38.1 mg/dL vs. LPS+Hes, 191.9 ± 31.9 mg/dL, *p* < 0.001; Figure 7A). In addition, renal mRNA levels of TNF-α, IL-6, and IL-1β were also reduced by hesperetin (TNF-α: LPS, 12.3 ± 1.0 vs. LPS+Hes, 3.8 ± 0.5, *p* < 0.001; IL-6: LPS, 11.5 ± 1.0 vs. LPS+Hes, 3.6 ± 0.7, *p* < 0.001; IL-1β: LPS, 12.7 ± 1.1 vs. LPS+Hes, 5.3 ± 0.9, *p* < 0.001; Figure 7B). To examine the effect of hesperetin on macrophage infiltration, sections were stained with an antibody against F4/80 [34,35]. IF staining showed that LPS-injected mice displayed a significantly higher number of F4/80-positive cells (Figure 7C,D). However, hesperetin treatment significantly inhibited macrophage infiltration (LPS, 11.8 ± 1.3 vs. LPS+Hes, 5.0 ± 1.2, *p* < 0.001; Figure 7C,D).

**Figure 8:**
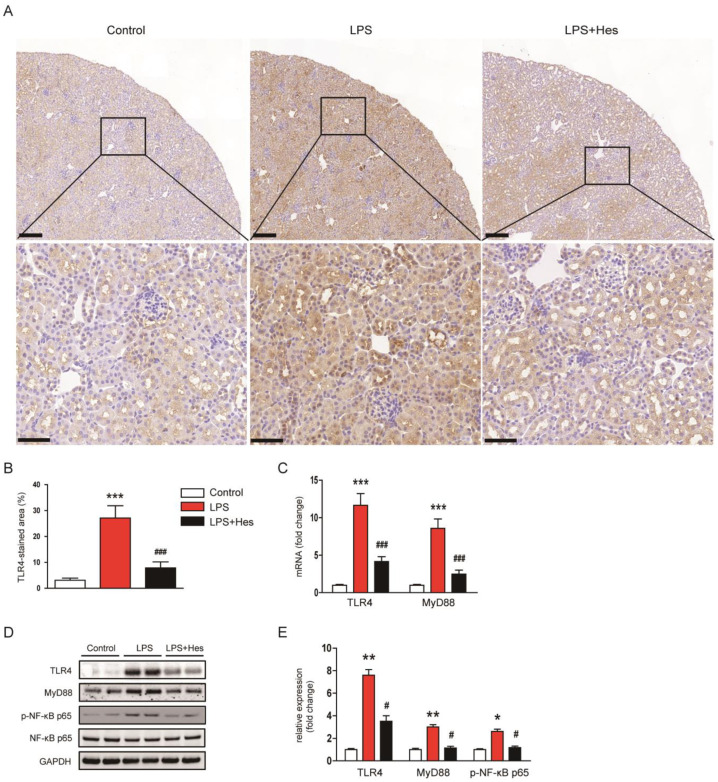
Hesperetin inhibited the Toll-like receptor 4 (TLR4)-MyD88-NFκB pathway. (**A**) IHC staining for TLR4. Scale bars: 150 μm (upper panel), 50 μm (lower panel). (**B**) Percentage of the TLR4-stained area per field. (**C**) Renal mRNA expression of TLR4 and MyD88. (**D**) Western blotting of TLR4, MyD88, and p-NF-κB p65. (**E**) Densitometric analysis of bands in (**D**). *n* = 8 per group. * *p* < 0.05, ** *p* < 0.01 and *** *p* < 0.001 vs. Control. ^#^ *p* < 0.05 and ^###^ *p* < 0.001 vs. LPS.

The TLR4 pathway plays a crucial role in the inflammatory response induced by LPS [7]. Therefore, we next examined the effect of hesperetin on the TLR4 signaling cascade to elucidate the mechanism underlying its anti-inflammatory effect. IHC staining for TLR4 revealed that LPS-injected mice showed a marked increase in the TLR4-stained area (Figure 8A,B). However, increased TLR4 expression after LPS injection was decreased by hesperetin (LPS, 27.1 ± 4.7% vs. LPS+Hes, 7.9 ± 2.3%, *p* < 0.001; Figure 8A,B). In addition, hesperetin reduced renal mRNA expression of TLR4 and MyD88 (TLR4: LPS, 11.7 ± 1.6 vs. LPS+Hes, 4.2 ± 0.6, p < 0.001; MyD88: LPS, 8.6 ± 1.2 vs. LPS+Hes, 2.5 ± 0.5, *p* < 0.001; Figure 8C). Their protein levels were also reduced by hesperetin (Figure 8D,E). Furthermore, hesperetin inhibited phosphorylation of NF-κB p65 in LPS-injected mice (TLR4: LPS, 7.6 ± 0.5 vs. LPS+Hes, 3.5 ± 0.5, *p* < 0.05; MyD88: LPS, 3.0 ± 0.2 vs. LPS+Hes, 1.2 ± 0.2, p < 0.05; p-NF-κB p65: LPS, 2.6 ± 0.2 vs. LPS+Hes, 1.2 ± 0.1, *p* < 0.05; Figure 8D,E).

## 3. Discussion

Hesperetin is a flavonoid mainly found in citrus fruits and has been known to possess antioxidant and anti-inflammatory activities [13,14]. Accumulating evidence suggests that this compound exerts beneficial effects on a variety of inflammatory diseases, including obstructive kidney disease and diabetic nephropathy [15,16,17,18,19,20,21,22,23]. In this study, hesperetin alleviated structural and functional renal injury in LPS-injected mice, as evidenced by the amelioration of histological tubular damage, attenuation of the brush border loss, downregulation of the tubular injury marker, and a reduction in serum creatinine and BUN levels. AKI can result from many types of causes, including hypoperfusion, sepsis, and exposure to nephrotoxins [36]. Sepsis has a complex and unique pathophysiology, making septic AKI a syndrome distinct from other types of AKI [8]. Similar to our findings, recent studies have shown that hesperetin attenuates cisplatin-induced histological abnormalities and renal dysfunction in rodents [24,25]. Cisplatin is a widely used chemotherapeutic agent with a high potential for nephrotoxicity [37,38]. Taken together, these results suggest that the renoprotective effect of hesperetin is not limited to a specific type and can act on AKI caused by various insults, including nephrotoxins and sepsis.

Oxidative stress plays a critical role in the pathophysiology of septic AKI [8.9]. Indeed, LPS injection induces ROS production in the kidney [39,40,41]. Because hesperetin is known to exert potent antioxidant activity [13,14], we assessed the effect of hesperetin on oxidative stress. In this study, hesperetin reduced the amounts of lipid (4-HNE and MDA) and DNA (8-OHdG) oxidation products in LPS-injected mice, indicating that hesperetin inhibits LPS-induced oxidative stress. This effect of hesperetin was accompanied by NOX4 downregulation. NOX4 is an enzyme that plays a critical role in ROS production and oxidative stress in renal diseases [42]. Numerous studies have reported the upregulation of NOX4 in AKI models caused by various insults including LPS [43,44] and cisplatin [45,46]. Yoo et al. showed that NOX4-mediated ROS production induces histological abnormalities and renal failure in LPS-induced AKI [47]. Therefore, the downregulation of NOX4 induced by hesperetin may be critically involved in its inhibitory action on oxidative stress. In addition, antioxidant enzymes play an important role in defense against LPS-induced AKI [48]. In this study, the primary endogenous antioxidant enzymes, catalase and MnSOD, were downregulated by LPS injection, but hesperetin significantly reversed the expression of these enzymes. Activities of catalase and SOD were also increased by hesperetin. GHS is an endogenous antioxidant that exerts a protective effect against oxidative damage [48]. Hesperetin attenuated LPS-induced depletion of GSH in the kidney. Similar to our findings, hesperetin inhibited antioxidant enzymes to suppress oxidative stress and inflammatory responses in various disease models [49,50,51]. Altogether, these results suggest that hesperetin inhibits LPS-induced oxidative stress via modulation of pro-oxidant and antioxidant enzymes.

Tubular cell apoptosis is an important pathological mechanism of septic AKI [8,9]. Previous studies have reported that a higher number of apoptotic cells were observed in LPS-injected mice [10,11]. In this study, we performed TUNEL assay to detect apoptotic cells. Hesperetin significantly inhibited apoptosis in LPS-injected mice. Cleavage of caspase-3 and PARP-1 was also attenuated by hesperetin. In addition, hesperetin reduced the protein levels of p53 and the mRNA expression of its transcriptional targets, PUMA and Bax. These results suggest that hesperetin attenuates LPS-induced apoptosis via inhibition of p53-dependent caspase-3 pathway. Consistent with these results, hesperetin inhibited apoptotic cell death in animal models of sorafenib-induced cardiotoxicity [52], doxorubicin-induced pulmonary toxicity [53], and acetaminophen-induced hepatotoxicity [54].

Cytokine overproduction and immune cell infiltration are hallmarks of septic AKI [8,9]. Rodents injected with LPS have been shown to exhibit increased serum and renal levels of cytokines and marked infiltration of immune cells into the injured kidney [55,56]. LPS triggers an inflammatory response by stimulating TLR4 and recruiting MyD88 in immune cells and renal tubular epithelial cells [7,8,9]. Eventually, TLR4-MyD88 pathway activates the transcription factor NF-κB. Accumulating evidence points to the essential role of the TLR4 pathway in the pathogenesis of septic AKI [57]. In this study, we found that hesperetin suppressed cytokine overproduction and macrophage infiltration in LPS-injected mice. LPS injection activated the TLR4-MyD88-NF-κB signaling pathway, but this pathway was significantly inhibited by hesperetin. Wang et al. reported that hesperetin alleviates LPS-induced acute lung injury in mice via the inhibition of the TLR4-MyD88-NF-κB pathway [20]. Zhang et al. also reported that hesperetin inhibited the polarization of microglia and exerted a protective effect in a mouse model of ischemic stroke by suppressing the TLR4-NF-κB pathway [58]. Overall, these results suggest that the beneficial action of hesperetin on LPS-induced cytokine production and macrophage infiltration may be mainly due to the suppression of the TLR4-MyD88-NF-κB cascade.

Our study has several limitations. First, the dose-dependent effects of hesperetin were not evaluated. Characterizing the dose-dependent effects of a compound can provide important insights into its efficacy and application. Second, the experimental design did not include a vehicle control group. Compared to an untreated control, a vehicle control can determine whether the vehicle alone causes any effects.

In conclusion, our data demonstrated that hesperetin exerts a protective effect against LPS-induced structural and functional renal injury. Hesperetin inhibited LPS-induced oxidative stress via modulation of pro-oxidant and antioxidant enzymes. In addition, p53-mediated apoptosis was alleviated by hesperetin. This compound also attenuated TLR4-dependent cytokine production and macrophage infiltration. Although future studies will be needed to further elucidate the mechanism of action of hesperetin, our findings suggest that it could be a potential therapeutic option for septic AKI.

## 4. Materials and Methods

### 4.1. Animal Experiments

Eight-week-old male C57BL/6N mice were acquired from HyoSung Science (Daegu, Korea) and were caged at a temperature of 22 ± 2 °C and a humidity of 55 ± 5% under a 12/12 h light–dark cycle. The mice were randomly divided into three groups: a control group (*n* = 8), LPS group (*n* = 8), and LPS+Hes group (*n* = 8). Septic AKI was induced using a single intraperitoneal injection of LPS (10 mg/kg; dissolved in normal saline). Hesperetin (50 mg/kg) or vehicle (DMSO; 1 mL/kg) was administered intraperitoneally 1 h after LPS injection. LPS and hesperetin were obtained from Sigma-Aldrich (St. Louis, MO, USA). The dose of hesperetin was determined based on a previous study investigating its renoprotective effect in mice [24]. Mice were sacrificed at 24 h after LPS injection. Blood samples were collected using cardiac puncture. The right kidney was fixed in 10% formalin for histological analysis, and the left kidney was frozen in liquid nitrogen for protein and mRNA analysis. Animal experiments were approved by the Institutional Animal Care and Use Committee of the Daegu Catholic University Medical Center (DCIAFCR-221007-27-Y).

### 4.2. Histological Examination, IHC, and IF Staining

Kidney tissues were fixed, dehydrated in graded ethanol, embedded in paraffin, and stained with PAS stain. Tubular injury was scored in 10 random cortical fields (×300) of PAS-stained sections. Tubular injury was scored based on the percentage of injured tubules: 0, 0%; 1, ≤10%; 2, 11–25%; 3, 26–45%; 4, 46–75%; and 5, 76–100% [59,60]. Tubular injury was defined as tubular atrophy, tubular dilation, tubular cast formation, sloughing of tubular epithelial cells, thickening of the tubular basement membrane, or loss of the brush border [59]. For IHC, after antigen retrieval, kidney sections were permeabilized and incubated in a blocking buffer. The sections were probed with primary antibodies against NGAL (Santa Cruz Biotechnology, Santa Cruz, CA, USA), 4-HNE (Abcam, Cambridge, MA, USA), F4/80 (Santa Cruz Biotechnology, Santa Cruz, CA, USA), or TLR4 (Santa Cruz Biotechnology, Santa Cruz, CA, USA). Then, the sections were incubated with a horseradish peroxidase (HRP)-conjugated secondary antibody. Slides were scanned and viewed using a slide scanner (3DHISTECH Pannoramic MIDI, Budapest, Hungary). For IF staining, deparaffinized sections were incubated with anti-8-OHdG antibody (Santa Cruz Biotechnology, Santa Cruz, CA, USA). After being washed, the sections were incubated with a secondary antibody conjugated with Alexa Fluor 647 (Thermo Fisher Scientific, Waltham, MA, USA). The FITC-labeled LTL (Vector Laboratories, Burlingame, CA, USA) was used for detecting the brush border of proximal tubules. Nuclei were stained with 4′,6-diamidino-2-phenylindole (DAPI). Slides were viewed and captured using a confocal microscope (Nikon, Tokyo, Japan). Quantification of positively stained area was performed using image analysis software (IMT i-Solution, Coquitlam, BC, Canada) in 10 random cortical fields (LTL and NGAL, ×400; 4-HNE and TLR4, ×300) per sample. The number of cells stained with F4/80 or 8-OHdG was counted in 10 random cortical fields (F4/80, ×400; 8-OHdG, ×1000) per sample.

### 4.3. TUNEL Assay

Apoptotic cells were detected using a TUNEL assay kit (Roche Diagnostics, Indianapolis, IN, USA) according to the manufacturer’s protocol. Briefly, kidney sections were deparaffinized, rehydrated, and permeabilized. After being washed, the sections were incubated in the TUNEL reaction mixture. Nuclei were stained with DAPI. Positive cells were counted in 10 random cortical fields (×600) per sample.

### 4.4. Biochemial Analyses in Blood and Kidney

Serum creatinine and BUN levels were determined using a biochemical autoanalyzer (Hitachi, Osaka, Japan). Renal MDA and 8-OHdG levels were analyzed using the MDA assay kit (Sigma-Aldrich, St. Louis, MO, USA) and the 8-OHdG ELISA kit (Abcam, Cambridge, MA, USA), respectively. Renal GSH and GSSG levels were analyzed using the GSH detection kit (Enzo Life Sciences, Farmingdale, NY, USA). Catalase and SOD activities were determined using colorimetric activity kits (Invitrogen, Carlsbad, CA, USA). Serum TNF-α and IL-6 levels were determined using ELISA kits (R&D Systems, Minneapolis, MN, USA). All assays were performed according to the manufacturers’ protocols.

### 4.5. Western Blot Analysis

Tissues were lysed in RIPA buffer. Proteins were separated with polyacrylamide gel electrophoresis and transferred to a nitrocellulose membrane. The membranes were incubated with primary antibodies against NGAL (Santa Cruz Biotechnology, Santa Cruz, CA, USA), NOX4 (Novus Biologicals, Littleton, CO, USA), cleaved caspase-3 (Cell Signaling Technology, Danvers, MA, USA), cleaved PARP-1 (Cell Signaling Technology, Danvers, MA, USA), p53 (Cell Signaling Technology, Danvers, MA, USA), TLR4 (Santa Cruz Biotechnology, Santa Cruz, CA, USA), MyD88 (Santa Cruz Biotechnology, Santa Cruz, CA, USA), NF-κB p65 (Cell Signaling Technology, Danvers, MA, USA), p-NF-κB p65 (Cell Signaling Technology, Danvers, MA, USA), or glyceraldehyde-3-phosphate dehydrogenase (GAPDH; Cell Signaling Technology, Danvers, MA, USA). Then, the membranes were probed with HRP-conjugated secondary antibodies. The blots were visualized using enhanced chemiluminescence reagents (Millipore, Bedford, MA, USA). Band densities were quantified using ImageJ software (National Institute of Health, Bethesda, MD, USA).

### 4.6. qRT-PCR

Total RNA was extracted with TRIzol reagent. cDNA was synthesized from extracted RNA using a reverse transcription kit (TaKaRa, Tokyo, Japan). The transcriptional level was analyzed using qRT-PCR with SYBR Premix Ex Taq II (TaKaRa, Tokyo, Japan) and primers (Table 1) in the Thermal Cycler Dice Real Time System III (TaKaRa, Tokyo, Japan). GAPDH was used as the reference gene, and relative levels of mRNA were calculated using the 2^−ΔΔCT^ method.

### 4.7. Statistical Analysis

Data are presented as the mean ± SEM. Statistical significance was analyzed with one-way analysis of variance (ANOVA) and Bonferroni’s tests. A *p*-value less than 0.05 was considered significant.

## Figures and Tables

**Table 1:** List of primers.

Gene	Primer Sequence (5′→3′)	Accession No.
NOX4	F: TGTTGGGCCTAGGATTGTGTT R: AGGGACCTTCTGTGATCCTCG	NM_015760
Catalase	F: CCATCGCCAATGGCAATTAC R: AGGCCAAACCTTGGTCAGATC	NM_009804
MnSOD	F: GTAGGGCCTGTCCGATGATG R: CGCTACTGAGAAAGGTGCCA	NM_0136671
PUMA	F: AGCAGCACTTAGAGTCGCC R: CCTGGGTAAGGGGAGGAGT	NM_133234
Bax	F: GGTTGCCCTCTTCTACTTT R: AGCCACCCTGGTCTTG	NM_007527
TNF-α	F: GTTCTGTCCCTTTCACTCACTG R: GGTAGAGAATGGATGAACAC	NM_013693
IL-6	F: ACGGCCTTCCCTACTTCACA R: CATTTCCACGATTTCCCAGA	NM_031168
IL-1β	F: CTGTCCTGATGAGAGCATCC R: TGTCCATTGAGGTGGAGAGC	NM_008361
TLR4	F: GTGCCAATTTCATGGGTCT R: AGCCTGGTGACATTCCAAGACG	NM_021297
MyD88	F: TCATGTTCTCCATACCCTTGGT R: AAACTGCGAGTGGGGTCAG	NM_010851
GAPDH	F: ATGGTGAAGGTCGGTGTGAAC R: TTGATGTTAGTGGGGTCTCGC	NM_008084

## Data Availability

The data supporting the findings of this study are available within the article.

## Sample Availability

Samples of the compounds are not available from the authors.
